# Thermal and Chemical Characterisation of Reprocessed PET: A Study on Commercial, Recycled, Bottle-Grade and Textile Blend

**DOI:** 10.3390/ma18184394

**Published:** 2025-09-20

**Authors:** Susana Gomes, Ana Pimentel, Maria José Monteiro, Andréa Marinho, Amanda Melo

**Affiliations:** 1 CENTITVC—Centro de Nanotecnologia e Materiais Técnicos, Funcionais e Inteligentes, Rua Fernando Mesquita, 2785, 4760-034 Vila Nova de Famalicão, Portugal; sgomes@centi.pt; 2 CITEVE—Centro Tecnológico das Indústrias Têxtil e do Vestuário de Portugal, Rua Fernando Mesquita, 2785, 4760-034 Vila Nova de Famalicão, Portugal; apimentel@citeve.pt (A.P.); mjmonteiro@citeve.pt (M.J.M.)

**Keywords:** textile, PET, recycling, sustainability, methanolysis

## Abstract

The increasing environmental concerns surrounding plastic waste have intensified recycling efforts, particularly in the textile industry, where poly(ethylene terephthalate) (PET) is widely used for sustainable material production. The growing use of recycled PET (rPET) in textiles has prompted the need for reliable analytical methods to detect and quantify rPET content. This study differentiates between virgin and recycled PET by simulating mechanical recycling through five reprocessing cycles of three distinct PET grades, assessing changes in crystalline structure, intrinsic viscosity, molecular weight, and specific degradation markers. Differential Scanning Calorimetry revealed bimodal melting behaviour in reprocessed samples, while intrinsic viscosity and Gel Permeation Chromatography indicated molecular degradation. Notably, the release of dimethyl terephthalate (DMT) and dimethyl isophthalate (DMiP) was consistently observed as a function of degradation. These markers were identified and quantified using High-Performance Liquid Chromatography (HPLC) and Gas Chromatography (GC), with GC offering higher sensitivity and lower matrix interference. This study demonstrates that DMT and DMiP are robust chemical indicators of PET degradation and recycled content. This analytical approach, combining thermal, rheological, and chromatographic techniques, provides a scientifically sound and potentially cost-effective basis for traceability systems, certification protocols, and regulatory compliance in sustainable textile production.

## 1. Introduction

The pervasive use of plastics in various industries, including packaging and textiles, has led to significant environmental challenges, with poly(ethylene terephthalate) (PET) being one of the most widely used polymers due to its low cost, durability and versatility [1]. In response, the textile industry, recognised as one of the most unsustainable sectors globally, has been adopting a circular economy approach, promoting recycling and waste recovery [2,3]. Among the various recycling methods, mechanical recycling is widely employed for PET waste, particularly from bottles, due to its low cost, simplicity, energy efficiency, and lower gas emissions compared to chemical methods. This method is also versatile for textile waste in the apparel industry [4,5,6]. However, the properties of recycled fibres depend on the tearing process and the original fabric composition, making them difficult to define precisely [7]. Despite these challenges, recycled PET (rPET) has emerged as a promising alternative to virgin PET, prompting extensive research and development in this field.

For example, the study performed by Massoud and Dsilva [6] demonstrated that converting rPET into fibres saves 20% of water, reduces carbon emissions by 79% and decreases energy consumption by 76% compared to the process using virgin PET. Although mechanical recycling offers several advantages, a significant drawback is the degradation of PET that occurs during the extrusion process. This degradation can take place when recycled PET is converted into pellets for further processing or during subsequent steps that involve remelting, such as the melt-spinning process [1]. To address these issues, several strategies have been adopted, such as blending rPET with virgin polymers, incorporating reinforcing nanoparticles, and, in some cases, using chain extenders [1,8,9]. Blending polymers enables the tailoring of properties to meet specific application needs, such as enhancing impact strength, reducing brittleness, and improving thermal resistance. For instance, post-consumer rPET was blended with polycarbonate (PC) to mitigate degradation effects and reinforce mechanical properties [10]. Another approach involved blending rPET from used water bottles with virgin fibre grade PET at different ratios (20, 40, and 70 wt%), improving thermal stability. Among these, the 70/30 rPET/virgin PET blend demonstrated properties comparable to those of the virgin polymer [11]. Beyond polymer blending, reinforcing fillers have also been explored to enhance the properties of rPET. Talc was incorporated as a reinforcing agent for rPET, making the polymer more suitable for injection moulding applications [12]. Another widely studied approach is the use of chain extenders to counteract the degradation that occurs during recycling and reprocessing. Studies have shown that using the epoxydic multifunctional oligomer Joncryl effectively stabilises the molecular weight of both virgin and recycled PET. Concentrations between 1% and 1.5% have been sufficient to prevent significant degradation over multiple reprocessing cycles, while increasing the concentration to 3% has resulted in a molecular mass increase of up to 20%, confirming its efficiency in maintaining polymer integrity during processing [13]. While these strategies have improved the mechanical and thermal properties of rPET, its performance is significantly affected by thermomechanical degradation during processing. Research has focused on the impact of multiple reprocessing cycles on the stability and performance of polymers. Badia et al. [14] investigated the effects of thermo-mechanical degradation on PET by simulating mechanical recycling through successive injection moulding cycles. The research highlighted that degradation, caused by chain scission, resulted in a reduction in molar mass, which in turn led to significant declines in PET’s thermal, viscoelastic and mechanical properties. Similarly, María López et al. [4] examined the degradation of PET through multiple extrusion cycles, comparing reprocessed samples with virgin and commercially rPET. Their findings revealed significant reductions in viscosity, molecular weights (Mn and Mw), and toughness in reprocessed PET, correlating with the number of reprocessing cycles. Additionally, reprocessing accelerated crystallisation, resulting in the development of three distinct crystal populations in reprocessed PET, compared to just one in virgin PET, along with increased stiffness and fragility. In a related study, Kuan-Hua et al. [15] investigated the effect of repetitive reprocessing on virgin PA6, revealing similar patterns of molecular degradation and changes in mechanical properties after multiple cycles.

The European Union has established stringent targets under its updated Packaging and Packaging Waste Regulation, requiring that at least 50% of all plastic packaging be recycled by 2025. Since PET is the most commonly used plastic in plastic packaging, many studies focus on enhancing the recyclability and properties of PET. However, research on quantifying rPET content in final products, especially textiles, remains limited [16]. Although certifications such as the RCS (Recycled Claim Standard) and GRS (Global Recycled Standard) encourage the use of recycled materials, they only confirm the presence, rather than the exact quantity, of recycled content, despite a significant ongoing effort to close this analytical gap [17]. Recent research has demonstrated that combining infrared (NIR/ATR-MIR) with chemometric models can reliably estimate rPET content in commercial bottles with an error margin of ±10% [18]. Meanwhile, Raman spectroscopy, combined with chemometric models, can accurately identify and quantify recycled PET in water bottles with up to 95% classification accuracy and a ±15% error margin [19]. A laboratory using the GB/T 39026-2020 standard plays a vital role in ensuring accurate identification [20]. This method involves methanolysis and swelling-extraction reactions, followed by analysis of the results with high-performance liquid chromatography coupled with a diode-array detector (HPLC-DAD) to identify PET degradation by-products. Although methanolysis is a widely used chemical recycling process for PET [21,22,23], it enables the controlled breakdown of the PET chain, releasing small molecules such as terephthalic acid (TPA), dimethyl terephthalate (DMT), and oligomers. The concentrations of these molecules can differ between rPET and virgin PET, making this method suitable for comparative analysis. Swelling-extraction involves swelling the macromolecular PET in a solvent to facilitate the extraction of small molecules. Regarding HPLC-DAD, it is a technique commonly used to separate, identify and quantify chemical compounds in a mixture, serving various analytical purposes. For instance, Sabina Ion et al. [24] have utilised this technique to determine the reaction products and substrate excess of the decomposition of BHET (bis(2-hydroxyethyl) terephthalate)), an intermediate product resulting from the PET degradation. Gas chromatography (GC) can also be employed to determine the products resulting from the degradation of PET. Kristýna Sovová et al. [25] investigated the degradation of PET at temperatures of 500 °C and 800 °C, where they detected, by GC-MS (gas chromatography-mass spectrometry), a total of 30 compounds resulting from the thermal degradation. This technique can be used alone or in conjunction with thermal desorption (TDS), where heating releases volatile and semi-volatile compounds into a gas stream for gas chromatography (GC). For instance, E. Duemichen et al. [26] investigated the decomposition products of polyamide 6,6 (PA 6,6) and polybutylene terephthalate (PBT), identifying 11 compounds for PA 6,6 and 9 compounds in PBT. Additionally, the authors concluded that the TDS-GC-MS method proved advantageous compared to other standard techniques such as thermogravimetric analysis coupled with Fourier transform infrared spectroscopy (TGA-FTIR) and thermogravimetric analysis coupled with mass spectrometry (TGA-MS).

This study investigates the effect of reprocessing on various PET grades (virgin PET, recycled PET, and bottle-grade PET) after five reprocessing cycles, simulating industrial mechanical recycling processes while minimising contamination from other plastic residues. Although many studies have explored PET recyclability and degradation mechanisms, a lack of standardised methods remains for identifying and measuring recycled content in final products, especially in textiles. This gap is significant due to the increasing demand for recycled PET and the risk of allegations of greenwashing. Consequently, this work aims to determine how molecular and thermal degradation signatures, along with specific degradation by-products, can act as indicators of reprocessing history. Structural changes were analysed using thermal and rheological tests, as well as molecular weight measurements. Degradation markers were evaluated using HPLC-DAD and TDS-GC-MS. The results of this investigation aim to support the development of reliable analytical tools and standardised certification protocols for verifying the recycled PET content, thereby promoting transparency and consumer confidence in sustainability claims.

## 2. Materials and Methods

### 2.1. Sample Preparation

#### 2.1.1. Mechanical Reprocessing

Commercial poly(ethylene terephthalate) (PETv) PET Selenis BR35 was obtained from Selenis Portugal S.A. (Portalegre, Portugal) in the form of pellets, with an intrinsic viscosity of 0.59 dL/g. Recycled PET (rPET) was provided by Evertis Ibérica S.A. (Portalegre, Portugal) in flake form, with an intrinsic viscosity of 0.75 dL/g. POLYCLEAR^®^ (Indorama Ventures, Bangkok, Thailand) supplied bottle-grade PET (PETg) with an intrinsic viscosity of 0.83 dL/g. The materials were dried in a Carbolite drying hopper (Carbolite Gero, Neuhausen, Germany) at 100 °C for 12 h and then at 150 °C for an additional 4 h. Five processing cycles were conducted for each material using a co-rotating twin-screw extruder (Rondol Technology Ltd., Stoke-on-Trent, UK), with modular spindles, a diameter of 21 mm, and a length-to-diameter ratio (L/D) of 25. The temperature profile along the extruder was set to 245/245/240/230/230 °C, from the feed zone to the die. To facilitate subsequent characterisation, the reprocessed materials were micronised using a Retsch PM100 planetary ball mill (Retsch, Haan, Germany). The milling process employed 3 mm diameter grinding balls in 500 mL and 250 mL jars, resulting in particles with an average size of 300 µm. Only the PETv, rPET and PETg, along with their samples from the third (PETv_R3, rPET_R3, and PETg_R3) and fifth reprocessing cycles (PETv_R5, rPET_R5 and PETg_R5), respectively, were characterised.

#### 2.1.2. Fabric PES Materials

Five fabrics, made from both virgin PET and recycled PET, were collected from different producers, revealing their basic composition. These samples were assigned a code and are summarised in Table 1, along with their respective compositions. In the textile industry, PET is commonly referred to as PES, which stands for polyester, a terminology widely adopted in fabric labelling and classification.

### 2.2. Methanolysis of Reprocessed and Fabric PET

Methanolysis of PET affords, in the presence of a catalyst, the production of DMT and ethylene glycol (EG). In addition to these methanolysis products, isophthalic acid (IPA) recurring units are transformed into DMiP. Concerning IPA, it is often added in the synthesis process to produce modified PET resins. However, unreacted IPA monomer in the process may remain in a PET product and pose risks to human health. Therefore, since it may pose a threat to human health, the control and detection of its content in the manufactured polyester fabric is significant [27].

The content of IPA was calculated according to Equation (1), where DMiP concentration is obtained by interpolating the peak area in the calibration curve, and the factor of 0.8556 is the ratio between the molecular mass of IPA (166.131 g mol^−1^) and the molecular mass of DMiP (194.184 g mol^−1^).

(1) $$Content\;IPA\;=\frac{DMiP\;concentration\;\left(mg\;kg^{-1}\right)\times 0.8556}{10000}\left(\%\right)$$

The depolymerisation was performed according to the standard GB/T 39026-2020 (Test method for the identification of recycled poly(ethylene terephthalate) (PET) fibre). In this method, 0.80 g of PET was depolymerised in the presence of a catalyst, in this case, zinc acetate, in methanol at 220 °C inside a stainless-steel reaction tube for 2 h. Afterwards, the reaction tube was immediately quenched in water at room temperature. The resulting supernatant was filtered using a poly(tetrafluoroethylene) (PTFE) filter (0.45 µm pore size) and refrigerated at 4–6 °C for at least 8 h. Then, the supernatant from the methanolysis was filtered and left at room temperature for further analysis, as reported by GB/T 39026-2020.

Zinc acetate was purchased from Tokyo Chemical Industry (Tokyo, Japan). High-purity methanol of HPLC grades, supplied from Fisher Scientific (Waltham, MA, USA), was used without further purification.

To ensure the accuracy and reproducibility of the results, methanolysis was carried out in duplicate for fabrics and triplicate for PET pellets.

### 2.3. Differential Scanning Calorimetry

The differential scanning calorimetry (DSC) technique was employed to investigate the effects of reprocessing cycles on the crystallisation and melting behaviour of reprocessed and fabric PET. The tests were performed on Mettler Toledo DSC 3+ equipment (Mettler Toledo, Columbus, OH, USA) under a constant nitrogen flow. Samples with an average mass of 8–10 mg were used. A heating-cooling-heating cycle was applied at a rate of ±20 °C/min within the temperature range of 25 °C to 300 °C. The degree of crystallinity (χ_c_) was calculated according to Equation (2), where ∆*H*_m_ is the melting enthalpy, ∆*H*_c_ is the enthalpy of crystallisation, *φ*PET is the fraction of PET present in the samples, and ∆*H*^0^_f_ is the enthalpy of fusion of 100% crystalline PET (135.8 J/g) [28].

(2) $$\chi_{c}\left(\%\right)=\frac{{∆H}_{m}-{∆H}_{c}}{\varphi_{PET}\times {∆H}_{f}^{0}}$$

The DSC curves were deconvoluted using Origin software (Origin version 2021, 9.8.0.200). This process is used to separate and identify the different thermal events, particularly in cases where the curve exhibits a bimodal trend, which is common during differential scanning calorimetry (DSC) analysis.

Before performing the deconvolution, the baseline of the DSC curve was adjusted and subtracted from the original curve to ensure that only the peaks of interest were analysed. In this software, the selected deconvolution method was Gaussian, which provided the best fit for the curve and the data. The next step was to choose the number of peaks, their positions, widths, and intensities, and fit them until the R-squared value (R^2^) was closest to 1. After fitting, the analysed peaks can be related to the thermal properties of PET as a function of the reprocessing cycles.

### 2.4. Gel Permeation Chromatography (GPC)

The reprocessed PET samples were weighed and allowed to swell in 1 mL of 15% *v*/*v* HFIP (1,1,1,3,3,3-hexafluoroisopropanol) in chloroform for 12 h at 30 °C, followed by 8 h at 45 °C, under continuous magnetic stirring. After that, the solutions were diluted in chloroform to achieve a 7% HFIP concentration and then filtered through a Branchia PTFE 0.22 μm filter (Sterlitech Corporation, Auburn, WA, USA) before injection.

The molecular weight of the PET samples was determined using Malvern Panalytical’s Omnisec system (Great Malvern, Worcestershire, UK), equipped with a refractometer (RI), right-angle light scattering (RALS, 90°), low-angle light scattering (LALS, 7°) and a viscometer detector. The measurements were performed with a PL HFIPgel guard and two PL HFIPgel 9 μm columns (Agilent Technologies, Santa Clara, CA, USA), 300 × 7.5 mm in series, plus a PS standard (105,000 g/mol) calibration. The two columns and detectors were maintained at 45 °C, while the autosampler was conditioned at 30 °C. The analysis was performed using a mobile phase consisting of 2% *v*/*v* HFIP in chloroform, with a flow rate of 1 mL/min, an injection volume of 100 μL, and a runtime of 40 min per sample. Each sample was analysed in duplicate. The data acquisition and calculation were executed using Omnisec software version 11.41 from Malvern.

Calibration standards were freshly prepared and injected on the same days as the GPC analyses. High-purity chloroform and HFIP, both of HPLC grade, were filtered before use and utilised without further treatment.

### 2.5. Intrinsic Viscosity

The intrinsic viscosity was determined using the “Standard Test Method for Determining Inherent Viscosity of poly(ethylene Terephthalate) (PET) by Glass Capillary Viscometer” [29]. This test method is used for determining the inherent viscosity of reprocessed PET soluble at a 0.5 vol% concentration in a 60/40 (*v*/*v*) phenol/1,1,2,2-tetrachloroethane solution, using a glass capillary viscometer. The procedure involves accurately weighing between 0.2475 and 0.2525 g of PET samples and dissolving them with 25 mL of solvent. The solution is stirred and heated at 110 °C for 15 min to ensure complete dissolution. Afterwards, an additional 25 mL of solvent at room temperature is added to the solution. Before measuring the flow time of the solution, the capillary viscometer must be calibrated using the pure solvent. To maintain consistency in viscosity measurements, it is crucial to control the temperature at 25 °C.

Mark Houwink’s equation, described in Equation (3), was used to calculate the molecular mass of the samples, after the solution’s intrinsic viscosity is determined:

(3) $$\left[\eta \right]=KM^{\alpha }$$

where K and α are empirical constants that depend on the pair polymer-solvent system and the temperature at which the intrinsic viscosity is measured, and M represents the weight-average molecular weight of the polymer. For conditions such as PET 60/40 (*v*/*v*) phenol/1,1,2,2-tetrachloroethane at 25 °C, the Κ and α values are described in Equation (4).

(4) $$\eta =4.68\times 10^{-4}({\bar{Mw})}^{0.68}$$

### 2.6. High-Performance Liquid Chromatography Coupled with a Diode-Array Detector

A HPLC-DAD technique was carried out to determine the DMiP and DMT in the samples.

The tests were performed on an Agilent 1260 Infinity II (Agilent Technologies, Santa Clara, CA, USA). The chromatographic separation was performed on a ZORBAX Eclipse XDB-C18 column (Agilent Technologies, Santa Clara, CA, USA) (4.6 × 250 mm internal diameter, 5 µm particle size). The column temperature was held at 30 °C. The injection volume was 10 µL. The mobile phase consisted of methanol (A) and a 90:10 (*v*/*v*) water/methanol solution (B). The flow rate was 1.0 mL/min, and the gradient elution programme was as follows: starting at 80% B, it decreased to 20% B within 15 min, followed by a 15–20 min linear gradient to 100% B, and then held at 100% B from 20 to 35 min. The eluent was monitored at 227 and 242 nm for detection.

For the determination of DMiP and DMT in the samples, about 2 mL of the supernatant resulting from methanolysis was filtered and analysed by HPLC-DAD. Simultaneously with the analysis of the samples, standard solutions were injected. The identification of DMiP and DMT was performed by comparing their retention times with those obtained by the injection of pure standards.

Quantification is performed using an external standard method, which involves constructing calibration curves with pure standard chromatographic reference compounds. For this, a mixed stock solution of 200 mg L^−1^ was prepared from DMiP and DMT that were dissolved in methanol and stored in the dark at refrigerator temperature (−18 ± 4 °C).

The stock solution was further diluted to prepare calibration standards used to construct the calibration curves for DMT (CAS 120-61-6), which was purchased as a standard reagent from Sigma Aldrich (Burlington, MA, USA), and DMiP (CAS 1459-93-4), which was obtained as a standard reagent from Toronto Research Chemicals (Toronto, ON, Canada). Five standard solutions with known concentrations ranging from 5 to 50 mg·L^−1^ were prepared in methanol on the analysis day to ensure stability and accuracy. These calibration standards were injected into the HPLC-DAD system to generate linear regression curves for each analyte. The method demonstrated excellent linearity across the tested range, with correlation coefficients (R^2^) of 0.999 for both DMT and DMiP, confirming the reliability of the quantification.

Quantification of DMT and DMiP in the PET samples was performed by interpolating the peak areas of each analyte into their respective calibration curves. The resulting concentrations are presented in Table S1. As discussed later, DMT was consistently detected in high concentrations across all PET types, validating its role as the main methanolysis product. In contrast, DMiP concentrations varied according to polymer type and reprocessing history, reflecting differences in IPA content and accessibility, which are explored in Section 3.

### 2.7. Thermal Desorption-Gas Chromatography-Mass Spectrometry

The content of DMiP and DMT in the samples was confirmed using TDS-GC-MS. In addition to analysing these two compounds, an initial pre-screening of several samples of PET pellets and fabrics was conducted in full scan mode. The mass spectra were obtained by collecting the data at a rate of 3.8 scans/sec over the *m*/*z* range of 25–400. The identification of the significant peaks was performed, and the compounds were identified through the National Institute of Standards and Technology (NIST20) [30].

For analysis of standards and the supernatant resulting from the methanolysis reaction, the Tenax TA tubes were spiked with 1 µL of the solution using a Tube Standard Preparation System (Gerstel GmbH & Co. KG, Mülheim, Germany). They were then flushed with helium at a rate of 100 mL min^−1^ for 2 min.

Samples were then analysed using a Markes International TD100-xr (Llantrisant, Wales, UK) linked to an Agilent 8890 gas chromatograph with a 5977C mass selective detector. The TD100-xr is programmed to start with a flow path at 150 °C and a pre-purge of 1 min at 45 mL min^−1^. Then, the sample tube is heated to 250 °C for 10 min with a flow of nitrogen (20 mL min^−1^), and the analytes are swept onto an electrically cooled focusing trap at 30 °C. Then, the focusing trap is rapidly heated to 300 °C (3 min), and the analytes are transferred or injected into the GC column.

The gas chromatography was fitted with an Agilent HP-5MS UI capillary column (Agilent Technologies, Santa Clara, CA, USA) (0.25 µm × 30 m, 0.25 µm film thickness). The oven temperature started at 40 °C (held for 5 min), followed by a temperature ramp of 3 °C min^−1^ to 90 °C, 5 °C min^−1^ to 160 °C, and 60 °C min^−1^ to 280 °C, held for 5 min using helium as the carrier gas. The ion source was maintained at 230 °C, and the quadrupole at 150 °C. The mass spectrometer was operated in SIM/Scan mode (*m*/*z* range of 25–400) under electron ionisation (EI). For DMiP and DMT calibration measurements, the fragmentation of mass-to-charge ratio (*m*/*z*) 163 was used for the examination.

Compounds were identified by comparing the retention times of chromatographic peaks with the reference compounds analysed under the same conditions. Quantification is performed using an external standard method, which involves constructing calibration curves with pure standard chromatographic reference compounds. A mixed stock solution of 500 mg L^−1^ was prepared from DMiP and DMT, which were dissolved in methanol and stored in the dark at −18 ± 4 °C. The calibration curve was created by preparing six standard solutions with known concentrations, ranging from 50 to 1000 ng. Calibration standard solutions were prepared in methanol on the day of analysis. For TDS-GC-MS, the IPA content was also calculated according to Equation (1).

## 3. Results and Discussion

### 3.1. Differential Scanning Calorimetry

The thermal properties of PETv, rPET, and PETg were analysed, including their behaviour after the third and fifth reprocessing cycles. The evaluated parameters, i.e., crystallisation temperature (T_c (cooling)_), glass transition temperature (T_g_), cold crystallisation temperature (T_c (heat)_), crystallisation enthalpy (ΔH_c_), melting temperature (T_m_), melting enthalpy (ΔH_m_), and degree of crystallinity (χ_c_), related to the second heating are presented in Table 2.

The reprocessing process leads to polymer degradation, which alters the crystalline structure and results in chain scission, thereby reducing the molecular mass. Small chains generally enhance segmental mobility, leading to variations in T_g_ [31]. In this study, however, the T_g_ remained unchanged. The lack of variation in T_g_ can be attributed to thermal lag and inertia caused by the heating rate, which influences the measurement of material properties and may obscure subtle structural variations in the material [32].

**Figure 1:**
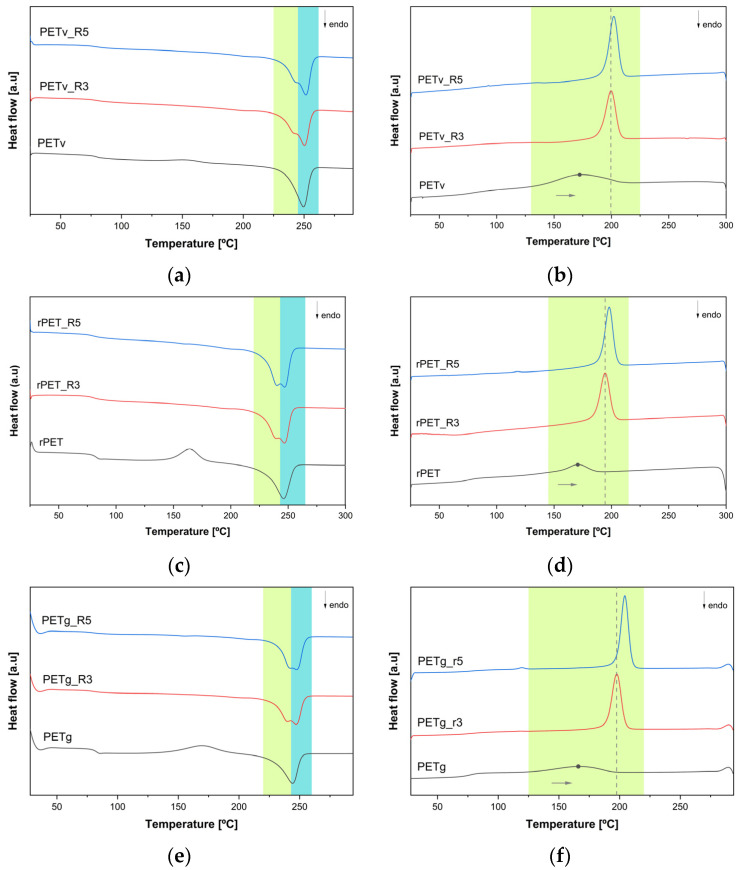
DSC analysis of PET samples during second heating and cooling cycles: (**a**,**b**) commercial PET; (**c**,**d**) recycled PET and (**e**,**f**) bottle-grade PET.

The melting temperatures of PETv, rPET, and PETg without reprocessing were 249 °C, 246 °C, and 244 °C, respectively, which are characteristic of each type of PET. While the PET without reprocessing shows one peak characteristic of T_m_, the samples that have been reprocessed exhibit a bimodal peak, as shown in Figure 1. In these cases, a secondary peak emerges at a lower temperature, whereas the primary peak remains centred around the original melting temperature of the corresponding unprocessed PET. The bimodal peak grew more pronounced as the number of reprocessing cycles increased. With this, an increase in melting enthalpy can also be observed as the new peak rises. This behaviour, also noted by [5,14], is attributed to the degradation of the PET and the distribution of crystals with varying sizes and lamellar thicknesses. Furthermore, in all reprocessed samples, the degree of crystallinity was higher compared to their non-reprocessed material, being particularly remarkable for the third and fifth reprocessing cycles of rPET and PETg. The short chains formed during reprocessing may serve as nuclei for crystallisation, facilitating the formation and growth of crystalline regions [4,11,14].

Figure 1 presents the DSC curves (second heating and cooling cycles) of virgin and reprocessed PET materials, highlighting the influence of multiple reprocessing steps on crystallisation and melting behaviour.

The cooling curve for the virgin PETv, rPET and PETg materials showed broader and flatter crystallisation peaks with lower enthalpy compared to the reprocessed samples, indicating a slower crystallisation rate. This characteristic is typical of materials with high molecular weight, which is essential for ensuring the transparency of high-quality bottles, as reported by [4]. After reprocessing the material, crystallisation occurred more rapidly as the crystallisation temperature during cooling (T_c (cooling)_) increased, highlighted with an arrow in Figure 1 and summarised in Table 2. Additionally, the cold crystallisation peak observed during heating (T_c (heat)_) was no longer detectable after multiple reprocessing cycles. The consistent thermal behaviour across all PET samples, such as the emergence of a second crystallisation peak during heating and the disappearance of the cold crystallisation peak, suggests that reprocessing exerts a similar influence across different PET grades.

Cold crystallisation typically results from the structural reorganisation of disordered or imperfect crystalline regions, which may occur due to rapid cooling during initial processing or the presence of residual amorphous domains between lamellae or within interspherulitic regions [4,33]. The absence of the cold crystallisation peak, along with the appearance of a second crystallisation event and an increase in crystallinity during the second DSC heating cycle, indicates that reprocessing-induced degradation promotes molecular ordering and decreases amorphous content. These findings suggest that thermal analysis could potentially be used to identify recycled PET content; however, further research involving a wider range of PET grades is needed to confirm the universality of these thermal signatures.

**Figure 2:**
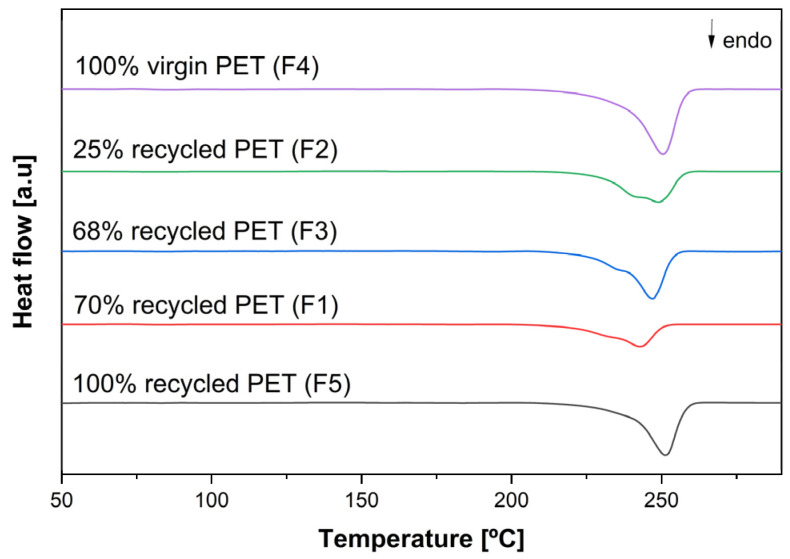
DSC curves for fabrics containing virgin and recycled PET during the second heating cycle.

In addition to the reprocessing samples, DSC tests were performed on PET fabrics with a known percentage of recycled material. The goal was to determine whether the material, in fabric form, exhibited the same behaviour or if deviations were present. Figure 2 shows the DSC curves from the second heating cycles of fabrics containing virgin and recycled material.

The graph presents the DSC curves of the second heating cycle for fabric samples containing varying concentrations of recycled PET. Given that a second melting peak was previously observed in the DSC analysis of reprocessed PET grades, absent in the virgin samples, it is reasonable to expect a similar behaviour in PET textiles containing recycled content. Consistently, the fabrics with known proportions of recycled PET exhibited a second melting peak, except for the sample made entirely from recycled PET. The absence of this peak in the 100% recycled PET fabric may be due to its processing method; instead of undergoing thermomechanical or chemical recycling, the material was re-spun into yarn, meaning the polymer chains tend to preserve their original thermal properties, similar to those of virgin PET [7]. As a result, while DSC analysis can indicate differences in crystallisation behaviour in recycled PET samples, this method alone is not yet sufficient to confirm the presence of recycled content. Moreover, transparency in the supply chain remains a challenge, as buyers often lack complete information about the recycling processes employed by suppliers.

#### Deconvolution Method

**Figure 3:**
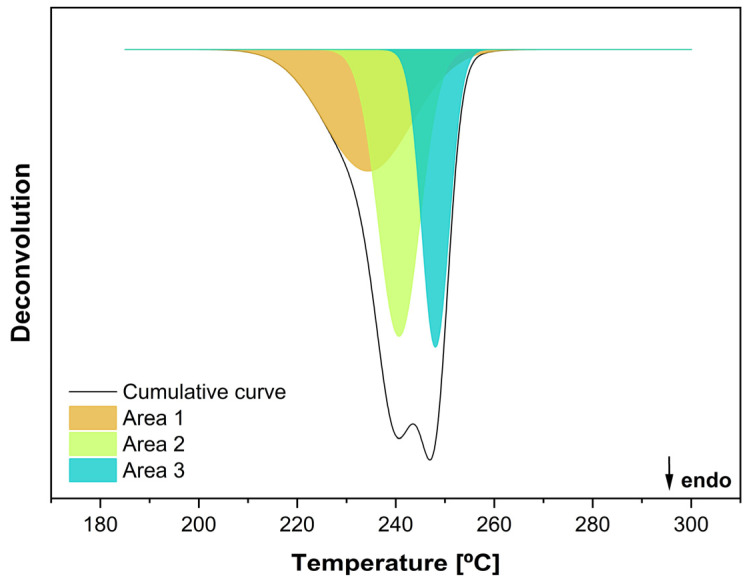
Deconvolution results for rPET after five reprocessing cycles.

During the second heating (i.e., after eliminating the material’s thermal history), a second melting peak was observed as the number of reprocessing cycles increased. Therefore, the second heating curves of the reprocessed material, the recycled material, and the bottle-grade material were deconvoluted to calculate and correlate the areas of each peak. According to the article by Kuan-Hua Su et al. [15], as the number of reprocessing increases, the area associated with the second peak also increases. To determine whether the samples behaved similarly, the areas related to the new peak were analysed to see if they increased with the number of reprocessings. To do this, the relevant areas of each sample and its respective replica were analysed. Peak 1, represented by Area 1, corresponds to a new melting event that emerges upon reprocessing. Peak 2, associated with Area 2, was introduced to improve the quality of the curve fitting. Finally, Peak 3, corresponding to Area 3, represents the original melting peak of the sample. To investigate the impact of reprocessing on the emergence of the second melting peak, the deconvolution procedure reported by López et al. [4] was employed. Figure 3 presents the deconvolution results for the fifth reprocessing cycle of rPET.

**Figure 4:**
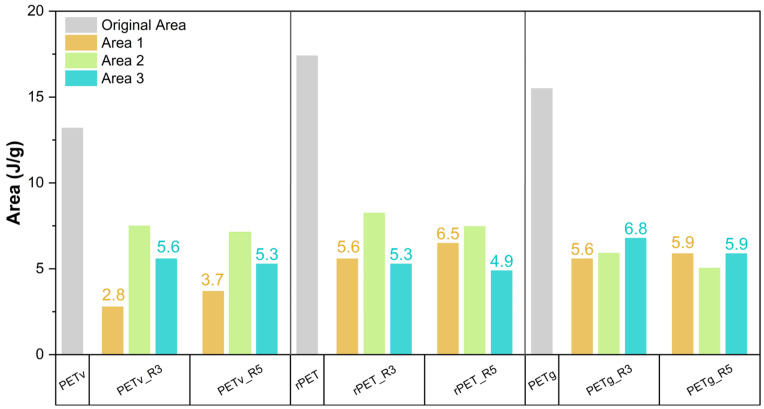
Average melting peak areas for commercial PET, recycled PET and bottle grade PET.

Figure 4 illustrates the average areas corresponding to the deconvoluted peaks for reprocessed commercial, recycled, and bottle-grade PET materials.

As shown in Figure 4, all materials exhibit the same trend, as the number of reprocessing cycles increases, area 1 expands, leading to new structural reorganisations. Area 3, representing the original peak after reprocessing, exhibits a decrease in all materials as the new peak emerges. This indicates that the polymer’s base structure and polymer chains are affected by reprocessing. Notably, as observed in the study by Kuan Hua Su et al. [15], this phenomenon occurs in all analysed materials subjected to multiple processing cycles.

Although it is not possible to quantify or verify the consistency of these changes, it is crucial to acknowledge this shared behaviour, which manifests regardless of differences in the chemical structures of the materials.

### 3.2. Gel Permeation Chromatography (GPC)

**Figure 5:**
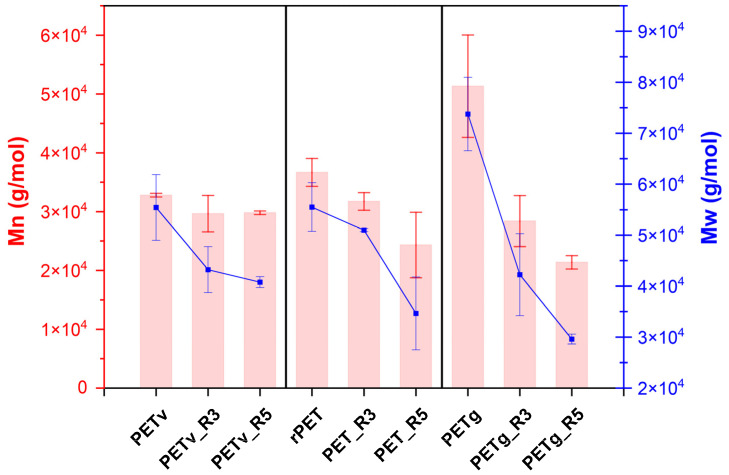
Molecular weight distribution of commercial PET, recycled PET and bottle-grade PET.

The molecular weight distribution of pristine PET samples (PETv, rPET, and PETg) revealed distinct differences in both number-average molecular weight (Mn, red bars) and weight-average molecular weight (Mw, blue line) as shown in Figure 5.

PETg initially exhibited the highest molecular weights, while rPET showed lower values, likely reflecting prior degradation in its recycling history [28]. Upon reprocessing, all PET grades demonstrated a reduction in Mn and Mw, indicating progressive chain scission caused by thermal and mechanical stress. This behaviour is notably apparent in the decline of the red and blue curves depicted in Figure 5. The degradation primarily results from thermal and hydrolytic cleavage of ester bonds during extrusion, resulting in shorter polymer chains [4,8,34].

To better understand the impact of reprocessing on molecular uniformity, the polydispersity index (PDI) and intrinsic viscosity (IV) were also evaluated (see Table 3). Initial PDI values for PETv, rPET, and PETg were 1.44, 1.45, and 1.51, respectively, reflecting relatively narrow molecular weight distributions in all grades. After reprocessing, PETv and PETg exhibited small reductions in molecular weight and intrinsic viscosity, accompanied by a slight decrease in PDI [35]. This suggests that although degradation occurred, it affected the molecular chains in a relatively uniform manner, possibly producing a narrower distribution of shorter chains.

Interestingly, PETv maintained a stable PDI after the third cycle (1.45) and showed a slight decrease after the fifth cycle (1.38), which may suggest resistance to thermomechanical degradation and a more homogeneous fragmentation pattern. In PETg, a gradual decline in PDI was observed with each reprocessing cycle, consistent with uniform chain breakdown. In contrast, rPET displayed a different behaviour: PDI increased from 1.45 to 1.48 after the third cycle and remained elevated thereafter. This trend indicates a broadening of molecular weight distribution, suggesting that chain scission was more random and non-uniform, likely due to the material’s previous processing history.

While the third reprocessing cycle generally caused less degradation than the fifth, the decrease in intrinsic viscosity observed at this stage was still significant enough to affect PET processability. The relatively stable PDI in PETv suggests a consistent degradation mechanism across the chain length distribution, whereas the fluctuation in rPET’s PDI confirms heterogeneous chain scission. Notably, PETv_R5 showed a slight recovery in intrinsic viscosity compared to PETv_R3, which may reflect less severe degradation in that cycle or experimental variability.

### 3.3. Intrinsic Viscosity

**Figure 6:**
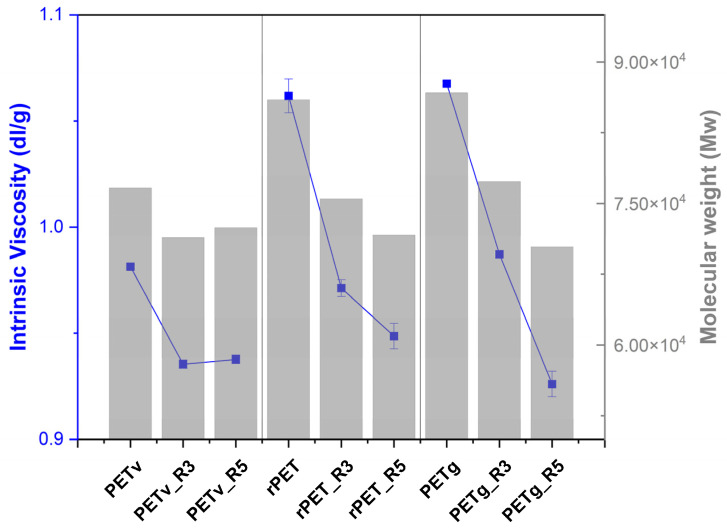
Average molecular weight and intrinsic viscosity of commercial PET, recycled PET and bottle-grade PET according to ASTM D4603.

To evaluate the effects of multiple reprocessing cycles, we measured the intrinsic viscosity (IV) of PET samples according to ASTM D4603 and estimated the molecular weight using the Mark–Houwink equation (Equation (3)). Table 3 and Figure 6 illustrate the different types of PET, which yield various results, mainly due to variations in their molecular weight and macromolecular structure. Furthermore, specific additives can be integrated into PET formulations according to their intended uses. For instance, reheat additives and oxygen barrier agents may be added to enhance performance. Nonetheless, these additives might also affect the hydrolysis reaction, resulting in alterations in intrinsic viscosity and molecular weight [36].

In this study, all PET samples exhibited a decrease in IV with repeated reprocessing, indicating ongoing chain scission, as confirmed by GPC analyses.

The initial intrinsic viscosities of rPET and PETg exhibited a similar trend of reduction, with initial values of 1.06 dL/g and 1.07 dL/g, respectively, decreasing to 0.94 dL/g and 0.93 dL/g after five cycles. This indicates that, despite having a similar initial molecular weight as shown in Figure 6, PETg is susceptible to additional degradation compared to rPET, regardless of its prior thermal history [37].

PETv, on the other hand, showed a more gradual decrease in IV, dropping from 0.98 dL/g to 0.94 dL/g over the five cycles. A slight increase in IV was observed for PETv in the fifth cycle compared to the third (PETv_R3), which may suggest less severe degradation during that specific cycle or could be due to experimental variability. Overall, the IV reductions align with GPC results, confirming a steady decline in molecular weight across all PET types and emphasising the cumulative effect of thermal and mechanical processing stress.

### 3.4. High-Performance Liquid Chromatography Coupled with a Diode-Array Detector (HPLC-DAD)

**Figure 7:**
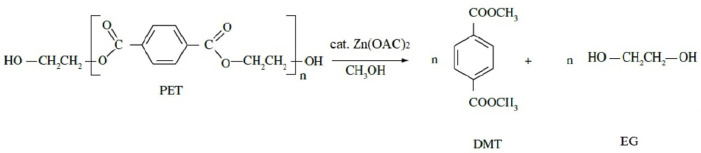
Reaction scheme of PET methanolysis with the catalyst.

To identify the degradation products of the three types of PET studied, methanolysis reactions were conducted. This chemical reaction facilitated the transesterification of PET; when PET reacted with methanol in the presence of the catalyst zinc acetate, DMT and EG were produced, as illustrated in Figure 7. Although IPA is not a mandatory component of the PET chemical structure, it is often present in the grade of PET used for bottles. During the methanolysis process, IPA undergoes esterification with methanol to form DMiP.

Due to this transformation, IPA is not directly detectable in chromatographic analysis. Instead, it is identified indirectly through its esterified derivative, DMiP, which acts as an indicator for its presence. This distinction in analysis is crucial for interpreting chromatograms. Furthermore, including IPA as a comonomer in the polymerisation reaction decreases the crystallinity rate, allowing it to be present within the polymer chain or as residual content [23,38].

The PET extract obtained from methanolysis was analysed using the HPLC-DAD technique, revealing the presence of DMT and DMiP monomers with retention times of 15.25 and 15.72 min, respectively. As expected, every PET grade includes DMT in its chemical composition. The results indicate no significant variation in DMT content across PET types, reprocessing cycles, or fabric samples, as shown in Table S1.

In contrast, DMiP was only detected in PET grades known to contain IPA as a comonomer, such as rPET, PETg, and the fabrics with recycled content from PET bottles, as shown in Table S1. In these cases, thermomechanical degradation during reprocessing leads to the formation of short chains and oligomers that readily react with methanol during methanolysis, facilitating the conversion of IPA units into DMiP. The efficiency of this conversion depends on methanol diffusion into the PET matrix, which the crystalline regions may hinder. Therefore, if IPA units are primarily located in the amorphous phase, their conversion into DMiP is expected to be more efficient [39].

In the PETg samples, both the third (PETg_R3) and fifth (PETg_R5) reprocessing cycles exhibited higher DMiP concentrations than the unprocessed material, with the increase being more pronounced in PETg_R5. This suggests that additional chain scission during this stage enhanced the accessibility of IPA units. In contrast, PETg_R3 had a lower DMiP concentration, likely due to partial degradation or restricted accessibility of IPA units at this intermediate phase.

For rPET, which has already undergone a prior recycling process, the DMiP concentration decreased in rPET_R3, followed by a slight increase in rPET_R5. However, both reprocessed samples remained below the original rPET value. This behaviour suggests that more severe degradation may lead to the volatilisation or breakdown of IPA-containing fragments into undetectable by-products, limiting their conversion into DMiP. This observation highlights a potential limitation in using DMiP as a reliable indicator for IPA content in highly degraded PET samples.

The percentage of IPA was estimated from the concentration of DMiP using Equation (1) and the results are presented in Table 4. The observed reduction in IPA content for the rPET samples (rPET_R3 and rPET_R5) is consistent with the progressive degradation promoted by thermal and mechanical stress during reprocessing in the extruder. In contrast, PETg samples showed a decline in IPA content after the third cycle, followed by a marked increase in the fifth cycle, indicating enhanced exposure of IPA units under more advanced degradation conditions.

The fabric containing recycled PET in its composition exhibited a significant DMiP concentration and consequently a higher IPA percentage, which may indicate that the recycled PET originated from recycled bottles [40].

### 3.5. Thermal Desorption with Gas Chromatography-Mass Spectrometry (TDS-GC-MS)

To corroborate and complement the findings obtained via HPLC-DAD, the samples were also analysed using TDS-GC-MS, which offers higher specificity for volatile and semi-volatile degradation products such as DMiP. As shown in Table S2, the degradation products DMT and DMiP were detected with retention times of 33.6 and 34.1 min, respectively. The consistent concentrations of DMT across different PET samples suggest a uniform terephthalic acid content, aligning with the standardised composition of PET polymers.

The IPA content was estimated from DMiP concentrations using Equation (1), and the results from HPLC-DAD and TDS-GC-MS are shown in Table 4. Although the absolute values differed, both techniques showed consistent trends in IPA content across PET grades and reprocessing cycles, reinforcing the reliability of DMiP as a specific analytical indicator of IPA content. Notably, TDS-GC-MS consistently yielded higher IPA values compared to HPLC-DAD. This discrepancy likely arises from the superior analytical specificity of TDS-GC-MS, which is more adept at detecting volatile and semi-volatile degradation products, such as DMiP. Furthermore, TDS-GC-MS is less affected by matrix effects that can hinder detection in HPLC-DAD. Taken together, the combined use of HPLC-DAD and TDS-GC-MS provided a more comprehensive assessment of PET degradation. HPLC-DAD enabled accurate quantification of soluble methanolysis products, while TDS-GC-MS offered superior sensitivity for detecting volatile species and degradation sub-products. Despite its analytical advantages, TDS-GC-MS requires more labour-intensive preparation and thermal desorption steps.

DMiP, formed by the reaction of methanol with IPA units, was identified at various concentrations in all grades of PET and in fabric samples that contain recycled PET from bottles. For example, the concentration was low for the virgin PETv samples, as expected, because IPA is typically added to bottle-grade PET and not to virgin resin formulations. In PETg, an increase in DMiP, and consequently IPA, was observed after the third (PETg_R3) and fifth (PETg_R5) reprocessing cycles, compared to the unprocessed material. This indicates that degradation during reprocessing enhanced the accessibility of IPA-containing units for conversion into DMiP. In contrast, rPET samples (rPET_R3 and rPET_R5) showed a reduction in IPA content with successive reprocessing cycles. This trend reflects the progressive degradation promoted by thermal and mechanical stress during extrusion, which may have led to the loss or volatilisation of IPA-derived structures.

The results for the fabric samples varied based on their composition. In the 100% virgin PET fabric (F4), only a minimal amount of IPA was found, which aligns with the lack of recycled content. The fabric containing 44% recycled PET (F2) also showed low IPA levels, as anticipated due to its limited recycled fraction. However, two fabrics with high recycled content, 100% rPET (F5) and 68% rPET (F3), unexpectedly displayed very low IPA concentrations. This may be explained by the recycling method employed, which was mechanical rather than chemical or thermomechanical and does not retain or enhance IPA content in the final product. In contrast, the fabric with 70% recycled PET (F1) exhibited a high IPA content, suggesting that the recycled PET in this sample likely originated from bottle-grade sources that include IPA as a comonomer.

## 4. Conclusions

In this study, commercial PET, recycled PET, bottle-grade PET, and textile samples containing recycled PET were analysed to assess the effects of reprocessing and the feasibility of detecting recycled content. All polymer samples, excluding textiles, underwent five reprocessing cycles, revealing progressive thermal and mechanical degradation as reflected in reduced molar mass, intrinsic viscosity, and increased crystallinity. These findings were supported by DSC and GPC analyses, which indicated recrystallisation and a consistent decrease in molecular weight.

Given the widespread use of IPA in bottle-grade PET, its presence was evaluated as a potential indicator in textile samples. PET depolymerisation via methanolysis allowed the conversion of IPA units into DMiP, which was then quantified. Both HPLC-DAD and TDS-GC-MS techniques were used, with TDS-GC-MS showing superior sensitivity in identifying degradation-related compounds.

Overall, the analytical approach proposed in this study shows high potential for identifying recycled PET from bottles in textile products. This work represents a significant step toward the development of trustworthy methods. However, further validation with a broader range of samples is recommended to establish a robust reference database for commercial applications.

## Figures and Tables

**Table 1:** Fabrics with different percentages of PES from other producers for depolymerisation.

Sample	Code
Fabric 1 (70% recycled PES, 30% virgin PES)	F1
Fabric 2 (44% recycled PES, 25% virgin PES, 28% viscose, 3% elastane)	F2
Fabric 3 (68% recycled PES, 29% viscose, 3% elastane)	F3
Fabric 4 (100% virgin PES)	F4
Fabric 5 (100% recycled PES)	F5

**Table 2:** Thermal properties of PETv, rPET and PETg and their respective reprocessing after the 3rd and 5th cycles.

Sample	T_c (cooling)_ (°C)	T_g_ (°C)	T_c (heat)_ (°C)	ΔH_c_ (J/g)	T_m_ (°C)		ΔH_m_ (J/g)	χ_c_ (%)
PETv	168 ± 1.20	80 ± 0.40	164 ± 7.86	1 ± 1.31	249 ± 0.12		34 ± 2.48	24 ± 0.92
PETv_R3	200 ± 0.06	79 ± 0.86	-	-	242 ± 0.39	249 ± 0.65	35 ± 2.68	26 ± 1.97
PETv_R5	203 ± 0.21	79 ± 1.05	-	-	243 ± 0.46	250 ± 0.17	35 ± 0.20	26 ± 0.14
rPET	162 ± 9.25	81 ± 0.24	162 ± 3.10	13 ± 8.84	246 ± 1.35		32 ± 1.78	14 ± 7.81
rPET_R3	195 ± 0.15	81 ± 0.31	-	-	240 ± 0.06	246 ± 0.06	34 ± 0.30	25 ± 0.22
rPET_R5	199 ± 0.00	80 ± 0.23	-	-	240 ± 0.06	246 ± 0.17	37 ± 0.08	27 ± 0.06
PETg	167 ± 1.61	81± 0.08	169 ± 1.62	14 ± 1.50	244 ± 0.61		33 ± 0.33	14 ± 1.35
PETg_R3	198 ± 0.18	81 ± 0.38	-	-	238 ± 0.00	246 ± 0.06	45 ± 0.54	33 ± 0.39
PETg_R5	205 ± 0.16	81 ± 0.07	-	-	241 ± 0.00	247 ± 0.14	49 ± 2.08	36 ± 1.53

**Table 3:** Intrinsic viscosity values of the reprocessed samples.

Sample	Polydispersity Index (PDI) ^1^	Intrinsic Viscosity (dL/g) ^1^	Mn (g/moL) ^1^	Mw (g/moL) ^1^	Intrinsic Viscosity (dL/g) ^2^	Mw (Mark–Houwink Method) ^2^
PETv	1.44	0.36	3.59 × 10^4^	5.22 × 10^4^	0.98 ± 0.001	7.66 × 10^4^
PETv_R3	1.45	0.33	2.67 × 10^4^	3.88 × 10^4^	0.93 ± 0.002	7.14 × 10^4^
PETv_R5	1.38	0.36	2.80 × 10^4^	4.24 × 10^4^	0.94 ± 0.002	7.24 × 10^4^
rPET	1.45	0.44	4.00 × 10^4^	6.03 × 10^4^	1.06 ± 0.008	8.60 × 10^4^
rPET_R3	1.48	0.27	2.91 × 10^4^	4.52 × 10^4^	0.97 ± 0.004	7.55 × 10^4^
rPET_R5	1.48	0.26	2.79 × 10^4^	4.00 × 10^4^	0.94 ± 0.006	7.16 × 10^4^
PETg	1.51	0.55	4.36 × 10^4^	6.60 × 10^4^	1.07 ± 0.001	8.67 × 10^4^
PETg_R3	1.48	0.44	2.68 × 10^4^	3.90 × 10^4^	0.99 ± 0.000	7.73 × 10^4^
PETg_R5	1.43	0.42	1.99 × 10^4^	2.86 × 10^4^	0.93 ± 0.006	7.04 × 10^4^

^1^ determined by GPC analysis. ^2^ determined by Standard Test Method for Determining Inherent Viscosity of poly(ethylene Terephthalate) (PET) by Glass Capillary Viscometer.

**Table 4:** Isophthalic acid content obtained by the two study techniques in PET pellets (pe) and fabric (fa).

Sample	IPA Content (%)	
	TDS-GC-MS	HPLC-DAD
PETv (pe) ^1^	0.060 ± 0.005	n.d. ^3^
PETv_R3 (pe) ^1^	0.053 ± 0.004	n.d. ^3^
PETv_R5 (pe) ^1^	0.0586 ± 0.0002	n.d. ^3^
rPET (pe) ^1^	1.92 ± 0.36	1.17 ± 0.08
rPET_R3 (pe) ^1^	1.64 ± 0.07	0.55 ± 0.03
rPET_R5 (pe) ^1^	1.08 ± 0.07	0.67 ± 0.02
PETg (pe) ^1^	0.61 ± 0.01	0.66 ± 0.04
PETg_R3 (pe) ^1^	1.10 ± 0.02	0.82 ± 0.17
PETg_R5 (pe) ^1^	1.38 ± 0.22	1.09 ± 0.25
70% recycled PET (F1) (fa) ^2^	1.88 ± 0.28	1.61 ± 0.06
44% recycled PET (F2) (fa) ^2^	0.40 ± 0.01	0.37 ± 0.02
68% recycled PET (F3) (fa) ^2^	0.53 ± 0.03	0.462 ± 0.005
100% virgin PET (F4) (fa) ^2^	0.12 ± 0.02	n.d. ^3^
100% recycled PET (F5) (fa) ^2^	0.12 ± 0.02	n.d. ^3^

^1^ Mean ± SD (Standard Deviation) (*n* = 3). ^2^ Mean ± SD (*n* = 2). ^3^ n.d.—not detected.

## Data Availability

The original contributions presented in this study are included in the article and Supplementary Materials. Further inquiries can be directed to the corresponding authors.

## Supplementary Materials

The following supporting information can be downloaded at: https://www.mdpi.com/article/10.3390/ma18184394/s1, Table S1: Concentration of DMT and DMiP present in the studied PET pellets (pe) and fabric (fa), (mg kg^−1^) obtained by HPLC-DAD and respective IPA content. Table S2. Concentration of DMT and DMiP present in the studied PET pellets (pe) and fabric (fa), (mg kg^−1^), obtained by TDS-GC-MS and respective IPA content.
