# Supplementary material for: Thermal and Chemical Characterisation of Reprocessed PET: A Study on Commercial, Recycled, Bottle-Grade and Textile Blend
Source: Materials (Basel). 2025 Sep 20;18(18):4394. doi: 10.3390/ma18184394 (PMC12471776; doi:10.3390/ma18184394)
Supplement: Supplementary file 1 [file materials-18-04394-s001.zip › materials-3716185-supplementary.pdf]

materials

MDPI

Supplementary Materials

Article

Thermal and Chemical Characterisation of Reprocessed PET: A Study on Commercial, Recycled, Bottle-Grade and Textile Blend

Susana Gomes [1], Ana Pimentel [2], Maria José Monteiro [2], Andréa Marinho [2,*] and Amanda Melo [1,*]

[1] CENTITVC—Centro de Nanotecnologia e Materiais Técnicos e Funcionais, Rua Fernando Mesquita 2785, 4760-034 Vila Nova de Famalicão, Portugal; sgomes@centi.pt
[2] CITEVE—Centro Tecnológico das Indústrias Têxtil e do Vestuário de Portugal, Rua Fernando Mesquita, 2785, 4760-034 Vila Nova de Famalicão, Portugal; apimentel@citeve.pt (A.P.); mjmonteiro@citeve.pt (M.J.M.)
* Correspondence: amarinho@citeve.pt (A.M.); amelo@centi.pt (A.M.)

Table S1. Concentration of DMT and DMiP present in the studied PET pellets (pe) and fabric (fa), (mg kg-1) obtained BY HPLC-DAD and respective IPA content.

Sample	DMT	DMiP	IPA content
PETv (pe)[1]	(1.56±0.25)E+05	n.d.[3]	n.d.[3]
PETv_R3 (pe)[1]	(1.2971 ± 0.0058)E+05	n.d.[3]	n.d.[3]
PETv_R5 (pe)[1]	(1.32 ± 0.12)E+05	n.d.[3]	n.d.[3]
rPET (pe)[1]	(1.57 ± 0.14)E+05	(1.363 ± 0.093)E+04	1.17 ± 0.08
rPET_R3 (pe)[1]	(1.29 ± 0.30)E+05	(6.39 ± 0.39)E+03	0.55 ± 0.03
rPET_R5 (pe)[1]	(1.34 ± 0.34)E+05	(7.80 ± 0.27)E+03	0.67 ± 0.02
PETg (pe)[1]	(1.52 ± 0.23)E+05	(7.70 ± 0.51)E+03	0.66 ± 0.04
PETg_R3 (pe)[1]	(1.59 ± 0.14)E+05	(9.6 ± 2.0)E+03	0.82 ± 0.17
PETg_R5 (pe)[1]	(1.47 ± 0.21)E+05	(1.28 ± 0.29)E+04	1.09 ± 0.25
70% recycled PET (F1) (fa)2	(1.54 ± 0.39)E+05	(1.879 ± 0.072)E+04	1.61 ± 0.06
44% recycled PET (F2) (fa)[2]	(2.08 ± 0.18)E+05	(4.29 ± 0.28)E+03	0.37 ± 0.02
68% recycled PET (F3) (fa)[2]	(2.39 ± 0.29)E+05	(5.404 ± 0.056)E+03	0.462 ± 0.005
100% virgin PET (F4) (fa)[2]	(1.19 ± 0.18)E+05	n.d.[3]	n.d.[3]
100% recycled PET (F5) (fa)[2]	(1.73 ± 0.18)E+05	n.d.[3]	n.d.[3]

[1] Mean ± Standard Deviation (SD) (n=3)

[2] Mean ± SD (n=2)

[3] n.d – not detected

Table S2. Concentration of DMT and DMiP present in the studied PET pellets (pe) and fabric (fa), (mg kg-1), obtained by TDS-GC-MS and respective IPA content.

Sample	DMT	DMiP	IPA content
PETv (pe)[1]	(1.717 ± 0.050)E+05	(7.03 ± 0.55)E+02	0.060 ± 0.005
PETv_R3 (pe)[1]	(1.188 ± 0.080)E+05	(6.25 ± 0.45)E+02	0.053 ± 0.004
PETv_R5 (pe)[1]	(1.478 ± 0.028)E+05	(6.850 ± 0.020)E+02	0.0586 ± 0.0002
rPET (pe)[1]	(1.56 ± 0.33)E+05	(2.25 ± 0.42)E+04	1.92 ± 0.36
rPET_R3 (pe)[1]	(1.439 ± 0.097)E+05	(1.917 ± 0.081)E+04	1.64 ± 0.07
rPET_R5 (pe)[1]	(9.57 ± 1.94)E+04	(1.261 ± 0.082)E+04	1.08 ± 0.07
PETg (pe)[1]	(1.36 ± 0.30)E+05	(7.106 ± 0.083)E+03	0.61 ± 0.01
PETg_R3 (pe)[1]	(1.12 ± 0.24) E+05	(1.283 ± 0.019)E+04	1.10 ± 0.02
PETg_R5 (pe)[1]	(6.71 ± 0.13)E+04	(1.61 ± 0.26)E+04	1.38 ± 0.22
70% recycled PET (F1) (fa)2	(1.08 ± 0.21)E+06	(2.19 ± 0.32)E+04	1.88 ± 0.28
44% recycled PET (F2) (fa)[2]	(2.88 ± 0.87)E+05	(4.664 ± 0.089)E+03	0.40 ± 0.01
68% recycled PET (F3) (fa)[2]	(1.502 ± 0.031)E+05	(6.25 ± 0.34)E+03	0.53 ± 0.03
100% virgin PET (F4) (fa)[2]	(1.35 ± 0.072)E+05	(1.39 ± 0.21)E+03	0.12 ± 0.02
100% recycled PET (F5) (fa)[2]	(1.20 ± 0.087)E+05	(1.45 ± 0.18)E+03	0.12 ± 0.02

[1] Mean ± Standard Deviation (SD) (n=3)

[2] Mean ± SD (n=2)

[3] n.d – not detected
